# Management of the Obstetrical Forceps

**Published:** 1871

**Authors:** C. C. P. Clark

**Affiliations:** Oswego, N. Y.


*Management of the Obstetrical Forceps.* By C. C. P. Clark, M.D., Oswego, N. Y. A Paper read before the New York State Medical Society.

In the fast growing and ambitious cities of the West, it may often be noticed that more attention is given to laying out and grading new streets, than to keeping in good condition the old thoroughfares of daily business. In these last, masses of refuse will be seen to hide a dilapidated pavement, while far out on the city's verge the surveyor and the paver are busy.

In our own art, if I mistake not, there is, in like manner, an eager pursuit of novelties, to the harmful neglect of principles and methods that are of daily application. I shall offer in this article some reasons for thinking that the rules and modes of using the obstetrical forceps have suffered this neglect, and I shall make some suggestions for their improvement.

Notwithstanding the indisputable fact that only a few experienced obstetricians acquire such dexterity and confidence in the use of the forceps as to resort to it freely, it may still be truly said that the whole armory of our art furnishes few instruments that are so useful in saving life and in lessening suffering. I think it furnishes none that are so capable of serving these ends. It fails of doing all it might do, because of the real or supposed difficulties and dangers that attend its use. Many an obstetrician, skilled and ready in everything else, is afraid of the forceps. Distrusting his own dexterity, and fearful of possible evils, he rarely or never resorts to this instrument. To the neophyte it is a terror.

I think the consciousness of my young readers and the recollection of their elders will give consent when I say that no operation in instrumental therapeutics is more dreaded by the novice than the delivery of a parturient by the forceps. I, for one, shall never forget the anxiety, the agitation, the sinking of the heart, the fear of failure, and, worse still, of the exhibition of incompetency, that preceded, nor the blind groping with the blades and the vain attempt to remember and apply the precepts of the books, that attended my early trials with this instrument.

Such repelling and unmanning terrors, and such fruitless efforts, be it observed, are doubly unfortunate, when, as in this operation, the crisis to be met is one that occurs to every practitioner, one that he must oftener meet alone, and one whose peculiar urgency is greatly augmented by the impatience, the anxiety and the expectation of friends, and by the suffering and often the danger of the patient herself.

In my own case I cannot be wrong in attributing these early fears and perplexities to faulty instruction. My purpose in this article is to show that these terrors do not legitimately arise from the character of the operation itself, nor are these difficulties inherent in it; but that they result from the erroneous way in which the subject is taught in books and in schools. I undertake to show that, in many of its most essential particulars, that teaching is defective and erroneous in substance, and in manner unnecessarily complex and obscure. A total want of uniformity in the rules laid down by different authors adds to the perplexity of the pupil.

In place of these obscure, complex, impracticable and discordant instructions, I undertake to give a set of rules that shall be simple and intelligible, that shall be applicable to all cases, and that shall thus rob the operation of its terrors, and make its practice, for obstetricians of ordinary intelligence and dexterity, easy and certain.

The points that I propose to touch upon are:

I. The danger that attends the use of the forceps.
II. The exigencies that call for the forceps.
III. The best kind of forceps.
IV. The position of the patient.
V. The law of application.
VI. The manner of introduction.
VII. Locking.
VIII. Slipping.
IX. Compression of the head.
X. Management in extraction.

On all of these points I shall venture to differ more or less widely from the received authorities, and I shall discuss only the particular matters in regard to which I thus differ from them.

I. Dangerousness of the Forceps.—Is the delivery of a parturient woman by the forceps attended with any considerable degree of danger to her? The general tone of our teachers and text books replies in the affirmative to this question. I shall never forget the earnestness with which that excellent and conscientious instructor, the late Prof. C. R. Gilman, used to impress upon the minds of his pupils the terrible dangers that attend the use of all midwifery instruments. It was his custom, in closing his lecture, to fling them from him with a somewhat theatrical show of terror and aversion, and to warn us in the most impressive manner of the fearful responsibility that accompanies their use. An innocent pupil was almost led to think that, in certain bad cases of labor, it was pretty much an even thing between letting the woman die a natural death, and twisting her womb off with the forceps, or letting her bowels out with the perforator.

On looking over authors, I find that these terrors are by no means peculiar to Professor Gilman. Cazeaux warns us of "lesions of the cervix and perforations of the vagina." He says: "There can be no doubt that the use of the forceps increases the danger of delivery." Churchill speaks of "laceration of the vaginal parietes, bruising the os-uteri," etc., etc. Blundell says: "In violent hands the long forceps is a tremendous instrument. Force kills the child, bruises the soft parts, occasions mortification, breaks open the neck of the bladder, crushes the nerves," etc., etc. So much is he afraid of wounding the soft parts, that he advises us always to count the pulse between the pains, to see whether we are killing our patient!

It was reserved for Dr. G. S. Bedford to reach the acme of terrorism, and to stir the imagination of his hearers and his readers with the most fearful pictures of ruin wrought by the forceps.

"The use of the forceps," he says, "is too often a scene of harrowing agony to the patient." He speaks of "fractured pelvic bones" and "disparted symphyses;" of "vesico-vaginal fistula," of "occlusion of the vaginal walls and the meatus urinarius," etc., etc., as common results after forceps delivery.

He pretends to support these statements by cases; but the reader who carefully examines these cases will see that they really give his representations no countenance. In the case, for example, which he gives on page 570, the history that he narrates in no way warrants him in attributing the calamitous result to the use of the forceps. It was probably due to the length—three days—and the severity of the labor, and would in all likelihood have been different if the forceps had been used in time. It was probably "masterly inactivity," and not the forceps, that did the damage. I believe that similar criticism will apply to almost all of the cases that are given in books to illustrate the dangers of the forceps; the premises will not warrant the conclusion, and, indeed, the history is generally too imperfect to warrant any conclusion. To me, with some observation and experience of my own, these terrible representations seem ridiculous and absurd. I affirm *that the forceps is not in any material degree a dangerous instrument* to the mother. In my own practice and observation, I have but once seen death follow a delivery by the forceps. In that case the fatal flooding was clearly due to exhaustion and uterine atonicity from too long delay of delivery; the application of the forceps and the extraction of the child was quick, easy and painless. I have seen some discreditable fooling, and a little cruelty, with the forceps, but I have never seen a case in which there was reason to attribute any injury of the parturient to its employment. When skillfully used it is not only harmless but painless. I never attend a patient whom I have delivered with this instrument without her asking for it again. The forceps is, indeed, a peculiarly innocent instrument. With its curved form and rounded edges it is almost incapable of mischief. It cannot cut, or puncture, or tear, or scrape. Neither can it bruise the soft parts, without the most stupid and reckless violence. As for its being pushed through the vaginal or uterine walls, it would be difficult or impossible to do it intentionally. Injury to the bony parts of the pelvis is equally out of the question.

The proportion of women that die after the use of the forceps is no evidence of its dangerousness; as well might we in the same way argue that bleeding or opium, or any other treatment, is dangerous in puerperal convulsions. Statistics are, therefore, of no value. It is obviously impossible to distinguish between the deaths that are caused by the forceps—if it is claimed that any one is so caused—and those that result from the conditions that called for them, or from other causes. The forceps is seldom resorted to, save in protracted and difficult cases, and in these a considerable mortality is to be expected from causes entirely independent of the instrument.

I will not deny that if the operator, following the instructions of the books, endeavors, obstinately and uncompromisingly, to introduce the long, double-curved forceps into the upper part of the pelvis, with its pelvic curve twisted from accordance with the anatomy of the passage thereto, he may injuriously bruise the maternal parts; nor will I deny that a similar or even greater evil may result if he imitate Dr. Elliott, (viz., his Obstetrical Clinique, passim), and wantonly and absurdly use such force as to "break" or "bend" a blade, "endanger the integrity of his instrument," "lay out his whole strength with braced feet," etc., etc. What I do claim is, that if he use ordinary anatomical knowledge, mechanical skill and common sense, the obstetrician cannot harm his patient with the forceps, and therefore the conscientious practitioner need never fear to try them. I think it would be difficult to find a single case in the books that, properly interpreted, even tends to prove the contrary. I have not found one. I am substantially supported in my opinion of the innocence of the forceps by Professor Simpson, as the reader will see by reference to his obstetrical works.

I shall not claim that this instrument is ordinarily *as harmless to the child as it is to the mother*. The features of the infant are in practice often temporarily, and sometimes permanently, disfigured by it, while the bones, and even the viscera of the head, have been sometimes fatally crushed. Dr. Elliott, in his "Obstetrical Cases," furnishes several instances of this. I do, however, claim that these injuries are entirely unnecessary, and proceed solely from want of skill in the operator. It is only when the grasp that the forceps gives is used for the purpose of compression, or to avoid slipping, that the instrument, when properly applied, can do any damage to the child's head. I expect hereafter to show that compression is never necessary or useful, and that, when properly applied, the forceps cannot slip.

The fear that seems to possess many obstetrical authors, of punching off the ears, or peeling the cranium of its scalp in passing the blades, is entirely absurd. Such an accident is substantially impossible. The beginner really need not have the bother of taking care of the child's ears added to his other troubles.

II. What are the Exigencies that call for the Forceps?—This question, in my opinion, admits of a simple and comprehensive answer. The occasion, the justification and the obligation, of using this instrument, are co-extensive and identical. Whenever, in a head presentation, with probable room for the head to traverse the pelvis, and with the os fully dilated or partly dilated and easily dilatable, the longer continuance of unaided labor involves danger either to the mother or to the child, or even a long duration of suffering to the mother, the forceps should be used. I go further; I hold that the forceps is justifiable sometimes in order to cut short the unnecessary protraction of those anxieties of the patient and her friends, that attend uncompleted labor, and even to save the time of the practitioner himself. I am well aware that in this opinion I widely depart from the maxim that authority sustains. Churchill, though reckoned an advocate and defender of the forceps, lays down the rule, in italics, for emphasis, that "they are to be applied in no case, till we are perfectly satisfied that the obstacle cannot be overcome by the natural powers with safety to the mother and the child." It is such a rule as this, causing perilous delay, that makes this instrument, in crude statistical tables, seem the means of death. I repudiate this rule. It is a rule that may be fitly followed by those who believe the operation to be difficult or dangerous, but it is not one for me, who think I find it as easy as the passing of a catheter, and as innocent as giving an enema.

As it is not my purpose to write a systematic treatise on the forceps, but only to touch upon those matters where I think the existing practice is erroneous, I shall not enumerate and discuss in detail the many items that are comprehended in the rule that I

have laid down, but shall content myself with some observations on certain supposed limitations of it that are to be found in the text books. Those limitations that hang on the supposed dangerousness of the operation I have already sufficiently discussed.

It is said that great violence of the pains contra-indicate the forceps, on the ground that a reinforcement of the expulsive power would be dangerous. This doctrine is entirely erroneous. The use of the forceps in such cases, in addition to its ordinary advantages, saves the womb from some part of that perilous violence of muscular action that, beside minor evils, sometimes threatens even its own integrity.

Neither is extreme resistance or rigidity of the soft parts a contra-indication. It is even an indication. If rigid perineal tissues be the obstacle, the danger of their laceration will be lessened by the forceps. The wedge-like form of the proximal end of the locked blades is an important aid in dilatation. It prepares the way. Meantime it diffuses the bearing of the uterine force along the longitude of the vagina, lessening its intensity at any point. On the other hand, so far as the resistance is due to the action of the perineal muscles, greater mechanical force in overcoming it is no way objectionable; and it can certainly be more cheaply furnished by the arm of the obstetrician than by the uterus of the mother.

Moreover, the experienced practitioner will remember that a majority of the cases of laceration of the perineum occur when, after long delay at that point, and many ineffectual pains, the uterus, as if vexed by the futility of its efforts, with one tremendous throe suddenly bursts through the obstacle. Reflex power, when repeatedly foiled, does thus accumulate. The forceps, by securing the steady progress of the head in some degree, obviates the danger.

Besides this, it is to be remembered that laceration of the perineum seldom or never occurs, save when there is a congenital deficiency of the elastic tissues of the vulva. This imperfect development may be hardly appreciable, or it may approach atresia. If it exist in even a slight degree, laceration of the parts is perhaps inevitable. Whatever the degree of danger may be, it will not be increased by the forceps unless the final delivery be wantonly and violently precipitated. The additional bulk made by the blades is next to nothing, and is more than balanced by the slight elongation of the head that almost necessarily attends their use. Meantime their wedge-like shape, beginning earlier the dilatation of the external parts, necessarily makes it more gradual and therefore safer.

Not a few authors declare that the use of the forceps should not be attempted when the head is above the superior strait. I can conceive of no good reason for this limitation. The operation under these circumstances is somewhat more difficult to the inexpert, and is sometimes impossible, but it is entirely free from the objection of peril. When the waters have escaped, tonic uterine contraction generally holds the head firmly against the inlet of the pelvis, with a larger or smaller segment of it engaged therein. In this condition a tolerably expert operator will have no great difficulty in grasping it with his instrument, and, if it be not detained by insuperable mechanical obstacles, delivery can be readily effected. On the other hand, when, from deficient tonicity of the uterine walls, or from the presence of a considerable quantity of amniotic fluid, or from both of these causes combined, the head is freely movable or floats above the pelvic brim, the attempt to apply the forceps will be of doubtful success. Turning is then the surer resource. This exigency may co-exist with hemorrhage, convulsions or other accidents calling for speedy delivery; but it can hardly obtain in any of the forms of dystocia proper, save in that in which there is so considerable narrowing of the pelvic brim as to make impossible any other means of delivery than embryotomy. The principal source, in my opinion, of the objection that many authors entertain to the use of the forceps when the head is in high situations, is in their utterly erroneous mode of applying the instrument. It is this that makes very many respectable authors oppose its use even when the head is already engaged in the superior strait, and needs but the touch of skill to cause it to finish its course.

III. What kind of Forceps should be used?—To the possessor of the long, double-curved forceps, the short forceps is utterly useless. Everything that can be done with the latter can be done with equal facility, safety and painlessness with the former. The obstetrician who sports both kinds, must be of a piece with the well-known gentleman who directed his carpenter to make in his garret door a big hole for the old cat and a little one for the kittens!

Equally unnecessary is it, in my opinion, to have several different forms of the long forceps. The editor of Prof. Simpson's Obstetrical Works reports him as saying, that "the more frequently he applied the forceps the more firmly he became convinced that one pair, of proper form, would answer for all forceps cases." (Vol. I, p. 442.) I entirely coincide in this opinion, and in the last fifteen years of tolerably extensive obstetrical experience have had occasion to touch but one pair. I am, accordingly, quite unable to appreciate the practice, in this particular, of Prof. Elliott, who, in his "Obstetrical Cases," reports himself as often trying several pairs in succession in the same labor. He seems to carry about with him as many tools as a carpenter or a burglar. This, of course, is not done for effect. He may safely trust more to his own skill and less to the skill of his instrument maker.

The exact shape of the blades and the mode of locking have been the subject of a great deal of attention, and of many modifications and supposed improvements. One would imagine, from the amount of ingenuity that has been expended on the subject, that accouchers were in pursuit of a key to open some intricate lock, and not of a pair of slender artificial hands to glide along a passage of well-known shape to a position that our own hands cannot reach, and to clasp there simply an ovoid body. All of the modes of locking are good enough; and I am inclined to think that the slight differences in the shapes of the modern instruments are not very material. The instrument that I use (and of course think the best that was ever made) is called in Paris "Dubois" forceps, and was made by Charrierre of that city. It is considerably longer, and somewhat more pronounced in its pelvic curve than Simpson's or Elliott's, and, in my opinion, is therefore better. I have not found it necessary to add to it the sliding guard of the latter inventor.

IV. The Position of the Patient.—The expert operator can apply the forceps equally well with the parturient on her side and on her back. The former position has this advantage, that, since women in this country are generally confined on the side, the arrangements for the operation in that position are less disturbing than in the other, and therefore less menacing to the imagination of the patient. This position also gives better access to the field of operation. I shall however recommend the other posture; for the reason that it better enables the practitioner to see, *with the mind's eye*, the anatomy, the mechanism, and the physics of the matter in hand. It is the position in which bodies are dissected, and in which we are in other ways most familiar with the female pelvis. In addition, this posture is symmetrical with the posture of the operator while operating—a fact which greatly facilitates both his conceptions and manipulations.

V. The Principle of the Application.—The grand question in the use of the obstetrical forceps, is, *whether the law of their application should have relation to the particular presentation of the head, or to the curve of the pelvic axis; to the anatomy of the child, or to the anatomy of the mother.* Shall we have regard to the theater, peculiar and nearly invariable in form, and definitely limited in extent, in which our operation is to be conducted, or to the posture in which the object to be seized may chance to lie? It is the former of these alternatives that the American, English and French authors that happen to be within my reach, unanimously adopt. Most of them say that the blades of the forceps should be applied "to the sides of the child's head," "parallel with the parietal bones." Even those who admit a less exclusive rule, substantially coincide with Prof. Bedford, who declares: "It is the position of the head that should determine the position of the blades." (Principles and Practice of Obstetrics, p. 558.) The same doctrine is taught by all the public lecturers on obstetrics that I have had the opportunity of hearing. In short, this is the principle that the American student finds everywhere laid down. It is not necessary to multiply quotations.

Prof. Simpson, it is true, appears to be partly aware of the absurdity of this rule. He says (Obstetrical Works, vol. I, p. 440), that "the application of the long forceps to the lateral or aural surfaces of the child's head at the upper strait, as described by Burns, Dewees, etc., and pictured by Churchill, is impossible in the very cases in which they are generally required;" for these reasons, among others, that "their pressure would greatly endanger the urethra and bladder in front," and "that they could no thus be placed in the axis of the brim, in consequence of the pressure of the perineum upon the instrument below." He makes like objections to the mode of application taught by Deleuyre, Davis and others, and illustrated in Ramsbotham. But when Dr. Simpson comes to lay down a method of his own, he falls into the same fundamental error as those whom he condemns. He directs the blades to be applied upon a certain diameter of the head. In other words, he teaches us to be governed by the *presentation*, and not by the maternal anatomy.

In my opinion this rule is entirely erroneous. I believe that the blades should simply follow the course of the utero-vaginal canal, and, when applied, should, in all cases, be in accord with the curve of the pelvic axis, regardless of the presentation. I believe that the presentation is not of the slightest consequence, and cannot be advantageously, or, in many cases, even innocently regarded. Wherever the sides of the head may be, the blades should be applied to the sides of the pelvis. I should perhaps hesitate in thus rejecting the traditional and uniform instructions of our authorities, did I not find it casually mentioned in Cazeaux, p. 802, that the method that I recommend is the practice of at least a part of the profession in Germany.

If the received doctrine on this subject be an error, it is a very grave error, and leads to very grave evils. The first of these is that it imposes upon the operator the necessity, as a preliminary step, of ascertaining the presentation. Even to the experienced practitioner this is not always easy; to the neophyte it is always difficult and uncertain. Knowing it to be so, he is never sure that he is handling his blades rightly. This doubt is a constant source of embarrassment and hesitation, and often makes him withdraw and introduce a blade again and again.

A far greater evil is that this doctrine necessarily makes the rules to be followed exceedingly complex: for the modes of introducing and applying the forceps must be as various as the presentations. Accordingly we find in all our authors a great variety of rules for the different presentations. Cazeaux gives special directions for each of eight vertex presentations when the head is at the inferior strait. Above that point, vertex presenting, he makes still other varieties of procedure. In face presentations he gives us similar changes in the modes of application.

Dr. G. S. Bedford has four variations in the application of the forceps at the upper, and four at the lower, strait. After these comes a miscellany of some half a dozen other modifications.

Dewees says, "the forceps should be applied to the sides of the head." He has four variations in this operation for the seven primary presentations that he counts, and still others for the more rare and difficult presentations.

Meigs lays down the same rule, "that the blades are to be applied to the sides of the head, and makes as many variations in the operation, some ten or a dozen, as consistency seems to him to require. His whole chapter on this subject is worth reading as an example of "confusion worse confounded."

Churchill says, that "at the brim of the pelvis the forceps may be applied in the transverse, the oblique, or the antero-posterior diameter, etc., etc., according to the presentations."

These references to popular authors, selected at random, are sufficient to show the extreme complexity of the rules for delivery by the forceps—as they are presented to learners at the present day.

It will be worth while for those who are curious on the subject to follow farther this comparison of obstetrical authorities. They will not fail to notice that, true to the native inconsistencies of error, the directions given in the different text books follow no common law or principle, but are various and conflicting in the utmost degree. The number of varieties in the mode of application is from three to more than a dozen. Some authors give different directions according as the head is at the upper or the lower strait, or between these points; others make no distinctions. Some make a difference in their instructions, according as the long or short forceps is to be used, others treat of both in the same words. So greatly unlike are their descriptions of the proper way of introducing and managing the blades, of the direction of the handles, etc., etc., in any particular presentation, that a reader would not suppose they were treating of the same case, or even the same subject.

What is the reason of these discrepancies and this confusion? Truth is always simple. The handling by learned and experienced men of a subject so long familiar to the profession as this has been, ought to exhibit the simplicity, uniformity and exactness of scientific truth. It exhibits in our leading authors none of these qualities. The cause is, that the principle with which they start, and on which their reasoning and descriptions are based, is essentially and totally erroneous. It is *not* the presentation that should govern the mode of application. However the head may present, the law that should govern the position of the blades is one and the same.

That law is, that the pelvic curve of the forceps shall follow and coincide with the utero-vaginal curve.

For what purpose, let me ask, is the pelvic curve given to the long forceps, unless it is to accommodate the shape of the instrument to the anatomy of the mother? It is only a single line of direction that this curve can fit, that is, the line of the pelvic axis, and it will bring the blades symmetrically against the sides of the pelvis, with the convexity of their pelvic curves following the bend of the sacrum. The curve of the sacrum and of the vagina, and the resistance of the floor of the pelvis—elements so powerful that in every labor we see them change the direction of the head by more than a quadrant of a circle—must be fully regarded, not only in the form of our instrument, but also in the position in which it may be placed in the utero-vaginal canal. Obvious as this would seem to be, I look in vain among the authors within my reach for the due acknowledgment of its importance.

I assert that till the head is actually at the outlet of the pelvis it is substantially impossible to apply the forceps in any other than the manner I have indicated. A slight deviation of the instrument toward an oblique diameter I admit to be possible, but its own shape and the laws of mechanics confine that deviation within narrow limits. How can you place the blades along the parietal bones when the plane of those bones makes an angle with that part of the pelvic axis in which the head is situated? Or how can you, without undue violence, lay them there when their pelvic curve must widely divert from and antagonize the curve of the maternal passage? The curve of the vagina still exists even when that canal is dilated to permit the passage of the head: can it be disregarded in the mechanics of forceps delivery? If we compare the distance, following the sacral curve, between the posterior commissure of the vulva and the posterior edge of the pelvic brim, with its anterior counterpart, it will be obvious that the blades, one following one line and the other the other line, cannot be brought squarely and symmetrically to embrace the head, without forcing their hands violently back to the very coccyx. Nor, when the head is at the superior strait, can it be done even thus. Nevertheless these are the virtual impossibilities that authors and lecturers, in the most matter-of-course way, call upon us to perform. They hardly ever suggest a difficulty or a doubt. Their language would make one think that the forceps can be played about in the female pelvis, with its pelvic curve bulging this way or that, as freely and easily as in an india rubber bag or in a barrel.

To cap the climax of absurdity, our professors illustrate their instructions on that most useless and preposterous of all human contrivances, called by Dr. Meigs, with unconscious appropriateness, "the Phantom." I well remember, as a pupil, spending hours over that effigy, learning, as I innocently supposed, to apply the forceps to the sides of the head when it presented in this, that and the other position. Nothing could be less like nature, and nothing, therefore, could be less instructive. It was like breakfasting on the morning fog. You might as well practice passing a catheter on the town pump.

I admit that when the head is at the pelvic outlet, the forceps may be applied to it in any of the diameters of that outlet. But even here the blades are best applied to the sides of the pelvis; for only thus will they be in symmetrical and easy relation to the maternal parts. The application of them in an antero-posterior position, or in a position approaching that, involves straining back the perineum in a painful and injurious manner, and threatens harm to the soft parts that underlie the pubis. For these evils this mode of application has no compensating advantages.

I shall be asked to reconcile the position I take in this matter with what authors represent themselves as doing. I prefer not to undertake to do this. When a man describes the application of the blades of the forceps antero-posteriorly at the upper strait, he describes what is impossible. Let me add that many times operators deceive themselves with regard to the direction in which the blades are passed; and many times authors, in their descriptions as well as in their maxims, blindly follow the beaten path.

If I am deemed guilty of unwarrantable audacity in speaking thus of our obstetrical authorities, I shall shelter myself behind the quotation that I have already made from Professor Simpson, If Burns and Dewees and Churchill describe impossible processes, and even sketch them for the engraver, as he says they do, why may not the most modern book-makers err in the same way? I am of the opinion that they do so err. I think, moreover, that it is entirely proper for any practitioner to repudiate authorities that are so utterly inharmonious as those that now bear sway in this matter of the application of the forceps.

Professor Elliott, though he follows the rest in instructing us to obey the presentation and apply the blades along the sides of the head, betrays the idleness of the rule when he says, page 300: "In difficult applications they will generally be applied over one of the oblique diameters of the fœtal head." Inspection of the head, after delivery, will show that they almost invariably lie upon the head in this manner, and almost never along the parietal bones.

An inspection of the cuts for illustrating forceps delivery that are to be found in treatises on midwifery, will show that the representation of impossibilities with which Professor Simpson charges Churchill is sometimes avoided by making the picture entirely inconsistent with the text. An application in an oblique diameter of the pelvis is described in the text, while the illustration of the forceps in position represents the instrument laterally applied, the locked handles being unmistakably in the plane of the transverse diameter of the pelvis, and resting squarely against the perineum. The fact is that a properly constructed blade of the long forceps, when once engaged between the head of the child and the wall of the vagina and pushed home along that canal, has, from its very shape, so strong a tendency to settle into the position to which alone its double curve is adapted, that only the perverse misdirection of ill-taught and violent hands can make it go astray. This is the reason why the young accoucheur, after repeated but futile efforts, has often found his instrument strangely and unexpectedly slip into its place just when he was despairing of success and had almost ceased to try.

It is easy, too, for older operators, .when the greater part of the blade is buried out of sight in the pelvis, to be deceived with regard to the direction it is taking, double curved as it is, and to erroneously believe that its whole course is on the line in which it began.

There are two ideas whose influence seems to have kept the minds of obstetric teachers fixed on always aiming to apply the blades of the forceps to the sides of the child's head. The one has relation to the safety of the child, the other to facilitating delivery by getting hold of the head endwise. In regard to the first, it is true that the blades so applied will "fit" somewhat better than when applied in any other diameter, but the head is so near a globe in its form that the difference in the fit or in the security of the hold is not material. The immunity of the child, in bone or feature or viscus, is never endangered by the proper use of the forceps. The obstetrician whose instrument disfigures the new-comer is a bungler whose only excuse is that he was taught in a bad school. Forcing the end or the edge of the blade into the child's tender flesh is a barbarous result. of that stupid idea in obedience to which we are told to *compress* the head in order to diminish its size or to prevent the instrument from slipping from its hold.

I shall, of course, admit that the head will pass easiest endwise; but I deny that the forceps can always be applied parallel with the long diameter of the head and along the parietal bones, and thus insure that facility. Whatever can be done by this instrument toward bringing the head into the most favorable position for delivery or toward directing its progress, will be best done by applying the blades symmetrically along the sides of the pelvis, fixing the head between them as in a frame, and, having it thus under control, giving to it whatever change of position or direction may be advantageous and possible. It will certainly be much easier to give your locked instrument a departure from mesial and symmetrical relation with the pelvis than to place the blades separately in that departure. It is safer, too; for the locked blade can move only with its fellow and with the enclosed head. Even in the most untaught or incautious hands its end or its edge can now do little harm. The locked forceps, applied in symmetry with the pelvis, may be regarded as an absolutely innocent instrument. With the enclosed head it may glide, or turn, or roll in the pelvis to some small extent, but it cannot jam, or cut, or tear, or bruise the maternal parts.

If it be true, as I have indeavored to show, that the obscure, complex, contradictory and multiform rules of the books are based on an erroneous principle, their abrogation is of the highest importance. Even if they were sound, their multiplicity and variety would make it impossible to remember them, while their obscurity would often make it difficult to understand them. When to this we add that their correct application presupposes that the practitioner can make himself sure of the presentation, which for most of us is often difficult, and for many of us sometimes impossible, it needs no argument to show that under their guidance the young obstetrician is indeed in pitiful straits.

Whose heart does not sink at the remembrance of his own first forceps case? Let the scene come back. His mind already possessed by the bugbear of the danger and difficulty of the operation, the novice first gropes and studies and sweats over the diagnosis of the presentation. Only partly sure of this, he next endeavors to find in his memory the special rule of the case. Is it strange, that, with such a mixed sea of authorities on this point as the books present, the endeavor is often vain? Nevertheless, he must go on. This is no time for delay or hesitation. He enters a blade. Practical difficulties now meet him. The blade will not go to its place. He is balked. Fears possess him. He doubts the correctness of his diagnosis, he doubts his memory, he doubts his skill. Confiding youth! it never occurs to him to doubt the soundness of his teachers. He begins to fear that he shall harm his patient; lacerations, ruptures—God knows what! —rise before his mind. He withdraws his instrument. He tries again, and perhaps, by chance, succeeds; or, failing a second and a third time, he sends for counsel, to find it, perhaps, as helpless as himself; or, no professional aid being within reach, he makes shielding excuses to the friends, of "contracted pelvis," or "slipping instruments," or "abnormal bulk of head," and resorts at last, more to be pitied than blamed, to the deadly perforator.

Is this not a true picture? And must not that young man be sustained by an exceptionally courageous heart who ventures to take his forceps in hand, save on the pressure of dire necessity, or of that public opinion of the lying-in-room which, direr still, calls upon him to show himself equal to every emergency, or to prepare to meet the sidelong glance of distrust, and even the pointing finger of contempt?

VI. The Manner of Introduction.—The principle of applying the forceps, according to the presentation, being thus proved to be illusory and impracticable, it remains to substitute for it a rule to which the obstetrician may safely trust in this important operation. Such a rule, simple to understand and easy to follow, is, in my opinion, not difficult to find. It is, that, in conducting the blades along the pelvic passage, and in grasping with them the head of the fœtus, we shall disregard entirely the presentation, and have regard only to the curve of the vagina and the contour of the pelvic cavity. The mind's eye must simply see a rounded body lying in the utero-vaginal canal, while the hand, obeying the anatomy of that canal, directs each blade of the forceps so as to pass around and embrace it.

Let this be established as the rule, and the operation becomes free from complications. It is no longer necessary to remember the endless variety of rules with which authors are filled. One simple law is in all cases to be followed, but two anatomical elements are to be held in the mind, and a uniform manipulation is to be executed.

It is, moreover, no longer necessary, as a preliminary, to ascertain the presentation. If there are those who assert that this is no bother, and who set me down as wanting the *tactus eruditus*, I shall simply ask them what they will do with the table given in Simpson's Obstetrical Works, ser. I, p. 414, according to which the ratio of occipito-posterior presentations to other presentations found by different experts, varies from 1 in 1336 to 1 in 3 or 4? or with the table given by the same author on the next page? I dare to assert, that if it be necessary to know the presentation before applying the forceps, the old rule of waiting till you can "feel the ears," is still a sound one.

I am well satisfied that the operation is, in fact, usually performed, sometimes intentionally, and sometimes unintentionally, on the principle that I have laid down above, and I confidently believe that my position will not need to be supported by authorities in the judgment of men of experience. But it is still necessary to introduce the doctrine into the books, and lay it before the generations of learners. It is for those that have the art of the forceps still to learn that I write.

If it be true, as indicated by Cazeaux, that this simple and easy rule of practice is somewhat generally followed in Germany, the fact will explain what we find in the statistics collected by Churchill. (Midwifery, p. 339, *et seq.*) It seems that the German practitioners there mentioned resort to the forceps nearly three times as often as do the English. It explains, too, why, as we are told by the same author, the Germans report a very small proportion of crotchet cases, and a very small fatality among children after the forceps.

Let us now approach the bedside of the patient, and proceed to perform what has properly been called the "obstetric miracle."

The first question that arises is: Which blade shall first be introduced? In regard to this little matter, singular obscurity and confusion will be found in authors. The individual blades are variously and loosely designated as the "upper and lower," "right and left," "male and female," "anterior and posterior," etc., and I nowhere find a uniform law of precedence clearly laid down. Such a law is, nevertheless, easily pointed out. That blade is to be entered first, which, when both are introduced and crossed, will be next the posterior commissure of the vulva. A moment's inspection of the lock will show which this is. It may most properly be called the posterior blade, and as the forceps are commonly constructed, is that one that must find its place in the left half of the pelvis.

Now, while the operator, with one or two fingers of his right hand supine, touches the scalp well back toward the sacrum, let him take this blade near its centre of gravity, with the fingers of the left, and holding it nearly perpendicularly, but a little inclined to his own left, slide it into the vagina along the palmar surface of his hand, till its extremity is engaged between the head and the maternal parts. From this point it is not the touch of the operator, but the imagination, the mind's eye, informed by anatomical knowledge, that must guide his motions. While he gently pushes along the blade, he must remember both the oval of the head and the bend of the vagina, and both the cranial and the pelvic curves of his instrument. He must remember, that while the point of his blade follows the utero-vaginal canal, it must so follow it as to bring its flat concave, not behind, but alongside the head, and its concave edge under the os pubis. In order to accomplish this double indication, the handle, as the point of the blade advances, must describe an intermediary between two curves. While it comes backward toward the operator and downward, it must also go outward toward his left and downward, and must describe in each of the two curves near a quarter of a circle. In other words, while the blade, in respect of its cranial curve, obeys the contour of the head, in respect of its pelvic curve it seeks the bend of the pelvic passage, and in order to adapt itself simultaneously to both, it must follow a spiral that contains them both.

In order that the young obstetrician may study the movement of his blade in detail and guide it most intelligently, it may be well for him, holding it as above described, at first to follow with its extremity simply the curve of the child's head, bringing the handle backward and downward, but not outward. As the blade advances deep into the pelvis, its convex edge will come to press against the right side of that cavity, its pelvic curve antagonizing in part the utero-vaginal curve, and the strain will resist its further progress. Now let him add to this movement obedience to the pelvic curve, by carrying the handle also outward and downward, and this convex edge will be turned gradually toward the sacrum, with whose curve its own contour is in harmony, and the before reluctant blade will glide along as if by instinct. Thus enlightened by watching his own progress and seeing the reason of it, the dullest disciple will hardly find difficulty in accomplishing the subsequent steps of the introduction. Whenever the onward progress of the blade is resisted, he will almost invariably find it due to an excess of one of the two above-described elements of the combined or spiral movement; and gentle trial of them separately will show him in which direction easy progress lies.

The double or spiral movement described is to be carried on till the handle is brought into the mesial plane of the body and firmly back against the perineum. The novice will be surprised at the length of the road he has to travel. Let there be no fear of passing the blade too far. There is no danger of doing this. After surmounting the convexity of the head, the end of the blade will necessarily approach the mesial line, and its impingement upon the body of the child will arrest its progress at the proper stage. From the opposite error great evils arise. From not carrying in the blades far enough it comes that they refuse to lock, and that their ends may be made to cut the scalp or gouge out the eyes, or may slip from their hold. The second or anterior blade is to be passed to its place in precisely the same way, *mutatis mutandis*, as the posterior blade.

The introduction of the hand into the vagina, as recommended by some authors (Bedford, for example), for the purpose of guiding the blade of the forceps, is an entirely superfluous piece of barbarity. The obstacle that often resists the onward motion of the blade is not a fold or cul-de-sac of the soft parts, whether of the mother or of the child. It is some false direction of the blade; and it usually and naturally results from the attempt of the practitioner to follow the erroneous rules laid down in the books. In my opinion there is no danger of punching a hole through the utero-vaginal cul-de-sac. When the edge of the os is beyond the reach of the finger, it has so far disappeared into the adjacent walls, that the extremity of the blade is in little or no danger of catching outside of it; and whatever danger there may be, is easily obviated by hugging a little the head of the child. The fear of punching off the ears of the child or of peeling its head, which are expressed by some authors, are not deserving of attention.

Most authors direct the first blade, when introduced, to be given to an assistant to be held in place. This is generally unnecessary. When the blade is pushed to its proper place and its handle carried well back, it will stay there.

VII. Locking—Probably there is not one of my readers, that, in his first essays with the forceps, did not have great trouble in making the blades lock. Even with experienced accoucheurs this is one of the most common and embarrassing of the difficulties that are met with. What solution of it is furnished by authors? None, or worse than none. The best they have to suggest is, to withdraw one blade or both, and try again. This is a sort of "scribe" rule, "cut and try" or "rule of thumb," that certainly does no credit to a learned and scientific profession.

Cazeaux recommends, in addition, that when one or both blades "turn outward," "the handles shall be grasped by the whole hand." What this means, unless it be an attempt to *force* them into place, it is not easy to see. If it means that, it is a vile rule. Dr. Bedford lucidly informs us, in page 588, that when the blades have embraced the head, the accoucheur will be able, "by judicious manipulation," to lock the forceps! This is highly instructive! What is "judicious manipulation"? The whole operation of the forceps is done by "judicious manipulation." Professor Meigs, page 558, speaks of "pushing" the blade this way and that, in order to bring it into a position to lock. These are fair samples of the guidance that the practitioner will find in the books in this important emergency. It is in default of better instruction that in our early trials we introduce and withdraw the instrument again and again, and, it may be, utterly fail at last. I cannot think that men who can furnish no better rules than these, fully understand the forceps. I believe that two simple precepts may be given that will almost invariably secure quick and easy success in locking the blades. These are: to push the blades far enough along, and to carry the handles far enough back against the perineum. The first step places the blades fairly upon the head, the second carries them back into the axis of that part of the utero-vaginal curve in which the head is situated; and, the blades being now symmetrical to each other, the handles must necessarily cross each other in the same plane, and will, therefore, lock. All ordinary cases of failure to lock, provided that the blades are laid anywhere in the neighborhood of their proper place, are due to deficiency in one of these particulars. These two steps of final adjustment, it will be observed, are but the continuation and completion of the process of applying the blades which I have already described; but their exactitude may properly be left till both blades are introduced and crossed, when if they do not lock, the fault almost always will be found to be in the incompleteness of one or both of these steps. In disproportionately roomy pelves, the course and position of the instrument not being normally controlled by the shape of that cavity and of the head, one or both of the blades will sometimes need to be slided towards the pubis. A deformed pelvis may make another exception to the sufficiency of the rules above given.

VIII. ARE THE FORCEPS LIABLE TO SLIP?—This is an important practical question. The reader will observe that in a considerable number of the cases in Dr. Elliott's "Obstetrical Cliniques" the forceps "slipped." Professor Meigs is so much afraid of this accident that he directs us to keep the finger against the head of the child in order to detect its incipience. In short, nearly all of our standard authors warn us against this danger. My forceps never slip, and in the face of these authorities I unhesitatingly assert, that in all ordinary proportions of the head and pelvis, with decent instruments correctly applied, the danger of slipping is wholly imaginary. Properly embraced between the blades, the head no way tends to slip from between the edges, either forward or backward; nor is there room for it to do so. Equally impossible is it for their ends to override the bulge of the cranium and let them come back to us empty. They must do this simultaneously if at all, and this will involve such wider separation of their bellies as the limits of the pelvic cavity do not admit. They certainly cannot slip over the head and come away without occupying more room in their void grasp than they would in bringing the head along with them.

When the forceps slip it is because the blades are not passed far enough to truly embrace the head. Their ends rest against it on or near its bulge, instead of reaching nearly or quite to the cervical region. This is the explanation of all the cases of slipping that I have ever seen, or can imagine. The instrument slips off because it has never been on. The question is important, not because the slipping of the blades can do any material harm, but because the apprehension of it is sure to lead to the wicked practice of violently

IX. COMPRESSING THE HEAD.—So far as the danger of slipping is concerned, I think I have shown that this is entirely unnecessary. The degree of compressing force that is necessarily used in grasping the instrument is all that is required, in any permissible exercise of extractive power, to make sure the hold of the forceps when properly applied, even in a disproportionately roomy pelvis.

But our authorities, moreover, with a good degree of unanimity, instruct us to compress the fœtal head in order to promote its elongation, and thereby facilitate delivery. Indeed, according to Prof. G. S. Bedford (Principles and Practice of Obstetrics, p. 575), "accoucheurs are divided as to whether the forceps acts principally as a compressor or an extractor." I can but regard this doctrine as erroneous and singularly unreasonable. The compression of an elastic body, while it diminishes one diameter of it, necessarily tends to increase all the diameters that are at right angles to the compressing force. Now it is obvious that there will seldom or never be need of reducing that diameter of the head which the blades of the forceps subtend, and which alone they can directly act upon. The contained must be less than the container, and the very fact that the blades have been passed around the head at the point where it has been stopped, is almost conclusive proof that in that diameter there is room enough. The obstacle, then, must be in another diameter of the head, and compression tending to increase that diameter, must tend to increase the obstacle. For example, it is seldom that a narrowing of any other than the antero-posterior pelvic diameter requires the application of the forceps at the superior strait. (*Vide* Simpson, *op. cit.*) But this diameter, as I have already shown, cannot be subtended by the forceps; they can only be applied in a diameter nearly or quite at a right angle with it; and whatever compression they are made to exercise upon the corresponding dimension of the head necessarily tends to increase that dimension that lies in the narrowed pelvic diameter, and thereby to increase the difficulty of the case. Whatever elongation of the head may be effected by compression is no compensation for this evil; it is obtained at the expense of an increase potentially of the very cause that demands the forceps. The resistance of the maternal parts to distention is the influence on which we must alone rely to promote elongation of the fœtal head.

Whoever is taught to use compression, either to promote security of hold or elongation of the head, will be pretty sure to overdo the matter, and will be in great danger of inflicting serious injury on the child. The iron levers give great power, and in the excitement of the moment that power is sure to be used. Such warnings as Churchill gives, "to limit the force used to what the head can bear without injury," are entirely useless. The benumbed and wearied muscular sense would deceive the best judgment. The injuries that the forceps are capable of inflicting, not simply on the child's scalp, but by compression upon the bones and even the viscera of its head, are well illustrated in Elliott's Obstetrical Cases, pp. 245, 246, 255—271. On p. 246, Dr. E. "changes his forceps in order to use compression with more effect." So far as the child was concerned, the "effect" evidently might have been spared.

X. Extraction.—It would hardly seem to admit of dispute that the extractive power of the forceps ought to be used in imitation of nature, and, accordingly, in the direction of the expulsive action of the uterine and abdominal muscles as modified by the lines of the pelvic passage. Nevertheless, for some incomprehensible reason, we are told by Prof. Bedford, that the force exerted by the obstetrician should be "one-third extractive and two-thirds lateral." This is also substantially the advice given by Prof. Meigs and other popular authors on midwifery. Such management of the forceps is not in imitation of nature. Nature does not see-saw or wriggle her burden along. Were the walls of the passage as dry, friable and inelastic as those of a post-hole, or were the fœtal head so rough and angular as to readily secure a bearing on those walls, this *prying it* out would not be unreasonable; but in reality its oval and gliding surface cannot be hastened along its lubricated and elastic road by working it from side to side. True it is, that, if our first efforts at moving the head along fail of success, we may very properly direct our subsequent tractions, tentatively, a little this way and that, distrusting the correctness of our judgment as to the exact law of the case, and thus learn to aid aright the vis-a-tergo. With this exception, based on the imperfection of human judgment, traction is the only function of the forceps.

It has seemed to me that the common conception of the movement of the fœtal head through the pelvis is somewhat erroneous in a very important particular. That traject is far more curvilinear than is commonly supposed. If we compare the distance traveled by the part that emerges from under the pubic arch with the distance that must be traversed by the part that follows the curve of the sacrum, we shall easily see that the head is rotated on the symphysis pubis almost as on a pivot. This must be borne in mind in our extractive efforts; and, at a point to be determined by our judgment, but earlier than is commonly supposed, they should be directed rather to *roll out* than to draw out the head.

The rules that I have thus laid down, especially those for the introduction and locking of the forceps, are sanctioned not alone by my own experience and judgment. More than one of my younger professional brethren have thanked me for my suggestions to them in this matter, and assured me that they had made easy to them an operation that had always before been embarrassing and difficult. I now submit these opinions more publicly to the profession. If my expression of them savors somewhat of disrespect for authority, I trust it will be pardoned. In practical matters of this sort we are far too much governed by authority. I believe I shall have the consent of the great body of our profession when I say that in many other particulars our literature needs a thorough overhauling. It needs it in order to the substitution of simplicity and clearness for complexity and obscurity, to the settlement of questions disputed but not disputable, and to the getting rid of rubbish that has the rottenness as well as the respectability of antiquity.

